# Insight into Antigenic Diversity of VAR2CSA-DBL5ε Domain from Multiple *Plasmodium falciparum* Placental Isolates

**DOI:** 10.1371/journal.pone.0013105

**Published:** 2010-10-01

**Authors:** Sédami Gnidehou, Leon Jessen, Stéphane Gangnard, Caroline Ermont, Choukri Triqui, Mickael Quiviger, Juliette Guitard, Ole Lund, Philippe Deloron, Nicaise Tuikue Ndam

**Affiliations:** 1 Institut de Recherche pour le Développement, IRD UMR 216, Mère et Enfant Face aux Infections Tropicales, Paris, France; 2 Université Paris Descartes, Paris, France; 3 Department of Systems Biology, Center for Biological Sequence Analysis, Technical University of Denmark, Lyngby, Denmark; 4 Unité d'Immunologie Structurale, Institut Pasteur, CNRS URA2185, Paris, France; 5 Institut des Sciences Biomédicale et Appliquées, Cotonou, Benin; The George Washington University Medical Center, United States of America

## Abstract

**Background:**

Protection against pregnancy associated malaria (PAM) is associated with high levels of anti-VAR2CSA antibodies. This protection is obtained by the parity dependent acquisition of anti-VAR2CSA antibodies. Distinct parity-associated molecular signatures have been identified in VAR2CSA domains. These two observations combined point to the importance of identifying VAR2CSA sequence variation, which facilitate parasitic evasion or subversion of host immune response. Highly conserved domains of VAR2CSA such as DBL5ε are likely to contain conserved epitopes, and therefore do constitute attractive targets for vaccine development.

**Methodology/Principal Findings:**

VAR2CSA DBL5ε-domain sequences obtained from cDNA of 40 placental isolates were analysed by a combination of experimental and *in silico* methods. Competition ELISA assays on two DBL5ε variants, using plasma samples from women from two different areas and specific mice hyperimmune plasma, indicated that DBL5ε possess conserved and cross-reactive B cell epitopes. Peptide ELISA identified conserved areas that are recognised by naturally acquired antibodies. Specific antibodies against these peptides labelled the native proteins on the surface of placental parasites. Despite high DBL5ε sequence homology among parasite isolates, sequence analyses identified motifs in DBL5ε that discriminate parasites according to donor's parity. Moreover, recombinant proteins of two VAR2CSA DBL5ε variants displayed diverse recognition patterns by plasma from malaria-exposed women, and diverse proteoglycan binding abilities.

**Conclusions/Significance:**

This study provides insights into conserved and exposed B cell epitopes in DBL5ε that might be a focus for cross reactivity. The importance of sequence variation in VAR2CSA as a critical challenge for vaccine development is highlighted. VAR2CSA conformation seems to be essential to its functionality. Therefore, identification of sequence variation sites in distinct locations within VAR2CSA, affecting antigenicity and/or binding properties, is critical to the effort of developing an efficient VAR2CSA-based vaccine. Motifs associated with parasite segregation according to parity constitute one such site.

## Introduction

Women suffering from pregnancy-associated malaria (PAM) develop antibodies that protect them and their offspring during subsequent pregnancies [1]. Protection against PAM is rapidly acquired as from the second pregnancy, and is associated with increasing plasma levels of PAM-specific anti-Variant Surface Antigen (VSA) antibodies. PAM parasites from distinct geographic areas specifically bind Chondroitin-Sulfate A (CSA) [2], [3], [4], and the immune response in pregnant women living in malaria endemic areas is highly directed against *var2csa* encoded PfEMP1 (*Plasmodium falciparum* erythrocyte membrane protein) protein [5], [6], [7]. Protective antibodies in PAM immunity are thought to recognize a relatively conserved antigen that mediates parasite binding to placental CSA, as plasma and parasites from pregnant women of different malaria endemic areas cross-react [5], [8]. Antibodies against VAR2CSA are sex-specific and parity-dependent, and high levels of such antibodies are associated with reduced consequences of PAM, making VAR2CSA a promising target for vaccine development [6], [7].

The VAR2CSA protein is a large antigenic molecule (350 kDa), exposed to host antibodies on the surface of erythrocytes [9], [10]. It has been shown that disruption of *var2csa* results in the loss of CSA adhesion ability of infected erythrocytes (IE) [11]. The VAR2CSA protein is structurally composed of six Duffy Binding-Like (DBL) domains. Several of these domains, including DBL5ε, have, to some extent, displayed affinity for CSA *in vitro* [12], [13], [14], [15] [16]. Antibodies raised against CSA-binding VAR2CSA domains have so far not been able to exhibit strong adhesion-inhibitory capabilities. However, antibodies raised against the recombinant DBL5ε domain amplified from a placental parasite, have been shown to bind native VAR2CSA expressed on the surface of *P. falciparum* IEs from placental isolates [16].

*Var2csa* is a polymorphic gene [17], and intra strain variability represents a great challenge for vaccine development. In a previous study, using genomic DNA from *P. falciparum* parasites from Senegalese women, the DBL5ε domain was found to be highly conserved among parasite isolates [18]. Mapping on a structural model revealed the localization of the DBL5ε identified polymorphic and some conserved regions in the exposed loops and helices [8], [18].

Although most VAR2CSA DBL domains contain conserved and polymorphic domain regions that can be targeted by surface reactive antibodies [8], conserved regions are most prominent in DBL3X, DBL4X and DBL5ε. This may explain why antibodies raised against DBL3X and DBL5ε recombinant proteins exhibited most cross-reactivity with heterologous parasites compared to antibodies raised against the other domains [19]. Interestingly, these antibodies (raised against a single variant of DBL3X or DBL5ε) cross-reacted with placental parasite isolates from Tanzania [20]. Moreover, human monoclonal antibodies produced by immortalized B cells from malaria-exposed pregnant women predominantly recognized DBL3X and DBL5ε [21], suggesting the natural acquisition of a specific immune memory to these VAR2CSA domains.

Together, these observations highlight that DBL5ε may represent an interesting target for vaccine development. Understanding the molecular basis controlling the broad and/or differential antibody recognition of this VAR2CSA domain may help define essential structural features of a potential interest in vaccine perspectives. The two main objectives of this study were: (i) To analyse the consequence of sequence variation in the VAR2CSA DBL5ε domain using the transcripts from a large panel of fresh placental parasite isolates and, (ii) to express and to characterize selected VAR2CSA DBL5ε variants from two parasite isolates. Novel conserved linear epitopes which are recognised by naturally acquired antibodies were found in the conserved regions of the DBL5ε domain and significant motifs were identified in the variable regions.

## Results

### Identification of significant sites in VAR2CSA DBL5ε sequences

**Figure 1:**
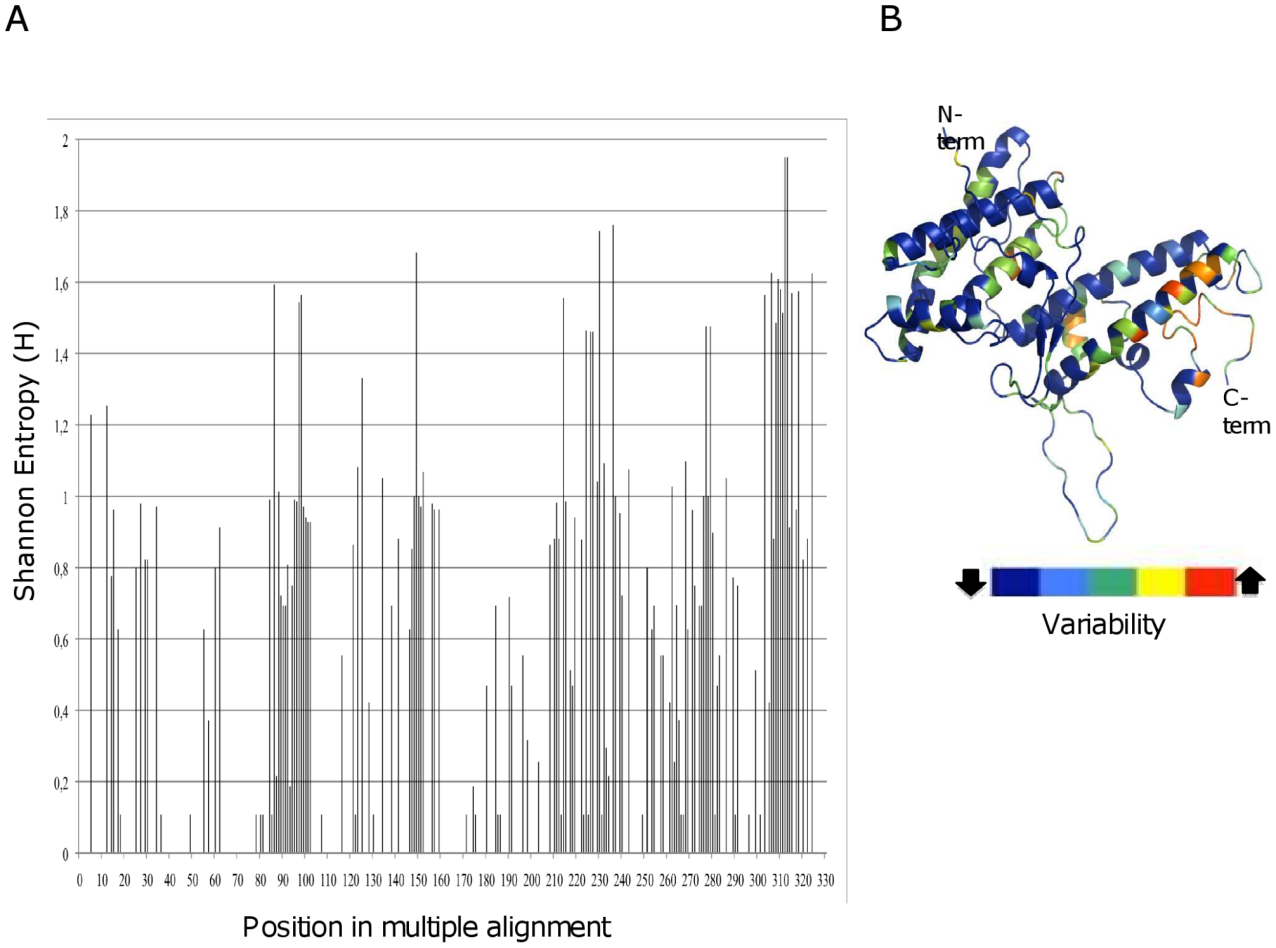
High conservation of DBL5ε-VAR2CSA sequences. (A) Plot of DBL5ε Shannon entropy (H): *H* = 0: Complete conservation, only one residue present at the given position. 0<*H*≤1: Considered highly conserved. 1<*H*≤2: Considered conserved. 2<*H*≤4.3 considered variable. (B) Three-dimensional model of DBL5ε showing the sequence variability. Heat-map colouring is dark blue (conserved) to red (variable).

**Figure 2:**
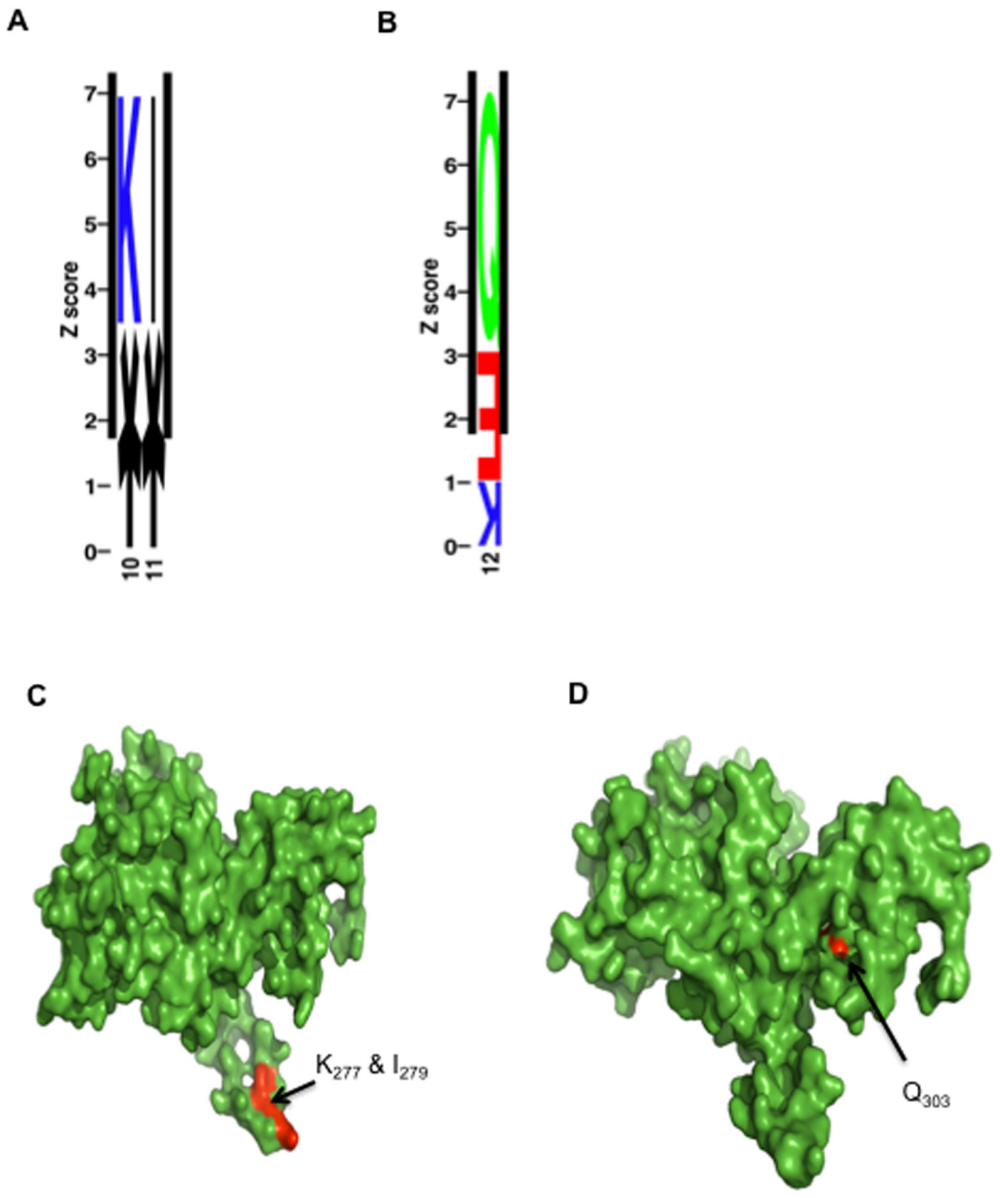
VAR2CSA DBL5ε patterns distribution. (A, B): Sequence logo showing the identified significantly distributed residues I, K and Q The sequence logo displays the residues present at each position, where at least one residue was identified as being significantly distributed with respect to associated numerical parameter. Each letter denotes a given residue and the height corresponds to increasing z-score. The residues are coloured according to: Acidic [ED]: red, Basic [RKH]: blue, Neutral [GNQSTY] = green, Hydrophobic [ACFILMPVW] = black. Numbers below each column denotes corresponding position in the multiple alignment. Letters positioned correctly are associated with high values and upside down letters with low. An asterisk denotes a deletion. It should be noted that in the sequence logos other residues appears (*, E, K), these are however not identified as significantly distributed (i.e. *p>0.05*). DBL5ε models showing the position of the identified significant residues (red), T_277_, I_279_ (C) and Q_303_ (D).

Figure 1 shows a multiple alignment of 70 VAR2CSA DBL5ε sequences (All sequence data are available at GenBank (http://www.ncbi.nlm.nih.gov/Genbank) under the accession numbers HM751723–HM751795) using cDNA from 40 placental parasites isolated at delivery from 39 Senegalese women [2], [15], [22] and one Tanzanian woman [20]. The var2csa region corresponding to DBL5ε plus Id5 (the non-DBL Interdomain sequence located between DBL5ε and DBL6ε) was cloned and sequenced. A total of 70 VAR2CSA DBL5ε sequences were obtained from these 40 placental parasites. The multiple alignment of DBL5ε sequences using the calculated Shannon entropy values show that the sequences consist of constant and variable blocks (Figure S1, Figure 1A). Conservation of 85% was obtained with DBL5ε and 80% when we considered DBL5ε plus Id5. The variability mapping on the DBL5ε structural model revealed that conserved and variable areas were located in loops and protruding helices (Figure 1B). In a previous study, it was found that VAR2CSA DBL3X sequence motifs can be linked to the parity of the infected women [15]. In order to assess such sequence variation behaviour in another highly immunogenic and conserved VAR2CSA domain, all DBL5ε sequences generated from cDNA of PAM isolates were analysed using SigniSite [23]. Analysis revealed that certain amino acids of VAR2CSA DBL5ε+Id5 sequences appear to be of particular interest. In the multiple alignment of all DBL5ε sequences, significantly distributed residues were identified at positions 277, 279, 303 (Fig 2A and 2B). High CSPG (Chondroitin Sulphate Proteoglycan) binding density is correlated with amino acid Q_303_ (p = 0.017). Homology modelling of DBL5ε-3d7 furthermore revealed that identified residues that were significantly different among groups were surface-exposed (Figure 2C, Figure 2D).

**Figure 3:**
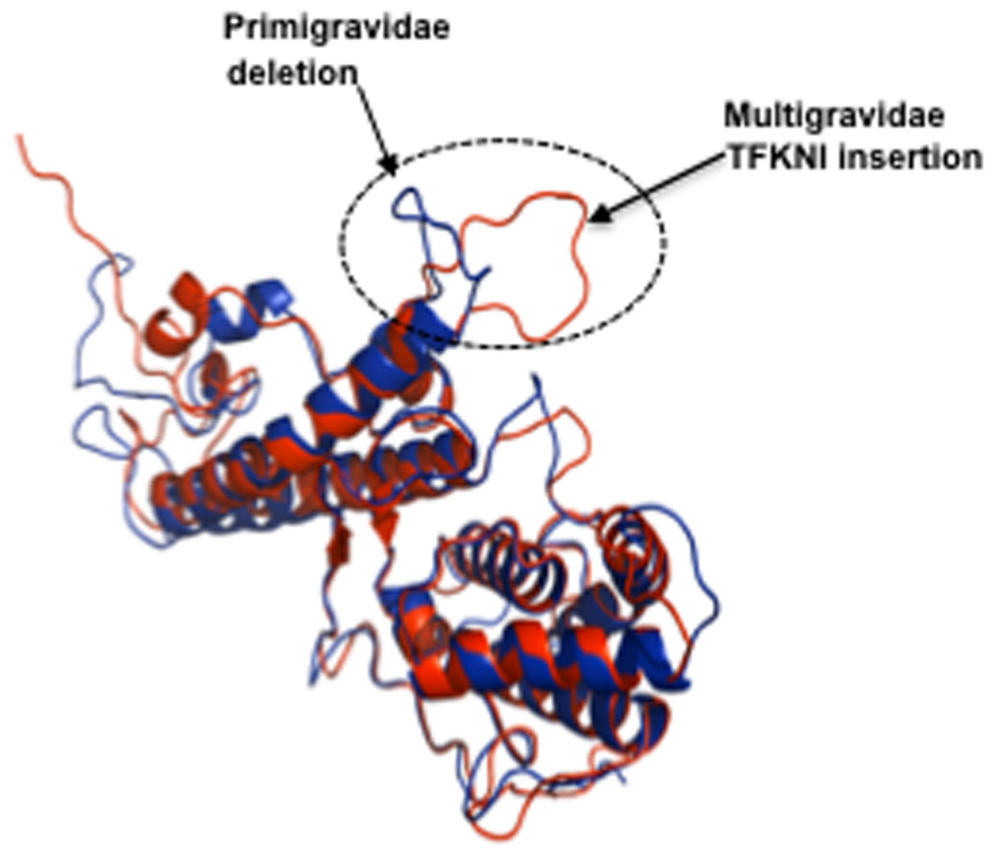
Mapping of VAR2CSA-DBL5ε signatures. Based on the identified region of interest and predominant motifs, two representative sequences were selected for homology modelling primigravidae CYK040 (deletion) and multigravidae CYK008 (TFKNI). Blue is CYK040 primigravidae sequence, red is CYK008 multigravidae sequence and dotted circle is deletion/TFKNI motif. The figure illustrates how the conformation of the region depends on the presence or absence of the TFKNI-motif. Using homology modelling, the motif is identified as being surface exposed and may thus alter the immunogenicity of the region.

From visual inspection of the regions around the amino acid residues found by SigniSite analysis in the multiple alignment of DBL5ε, distinct motifs were identified when comparing sequences from primigravidae and multigravidae. Motifs VFNNA, gap, TFKNI were identified in the area spanning amino acids 275 to 279 and EDTKQ, EYTGN and QYTGN were defined between the amino acid 303 and 313 (this area is located at the end of DBL5ε and in Id5). These patterns have a differential distribution according to parity. Indeed, gap, EDTKQ and EYTGN motifs were predominantly found among samples from primigravidae (p = 0.02, Fisher's exact test) whereas TFKNI and QYTGN were mainly or exclusively found in multigravidae (p = 0.013). These patterns clearly discriminate parasites infecting multigravidae and primigravidae women. At the level of sequence types obtained from each sample, DBL5ε sequences expressing gap, EDTKQ and EYTGN signatures were found mostly in primigravidae (p = 0.036) while those expressing TFKNI (p = 0.0019) and/or QYTGN preferentially infect (p = 0.038) multigravidae (Table 1). Interestingly the TFKNI motif was also associated with high maternal age and low placental parasite density (data not shown). The VFNNA motif was found in primigravidae as well as multigravidae without significant bias in its distribution. From the mapping of TFKNI and deletion motifs on the DBL5ε structural model from multigravidae CYK008 sequence and primigravidae CYK040 respectively, it can be hypothesised that TFKNI insertion can cause a conformational change of the domain structure (Figure 3).

**Table 1:** Signatures in DBL5ε domain of VAR2CSA expressed by placental parasites.

Category	Parity	VAR2CSA DBL5ε motifs					
		VFNNA	Gap	TFKNI	EDTKQ	EYTGN	QYTGN
**Samples**	Primigravidae (n = 16)	6	12^a^	1	7^a^	6^a^	0
	Multigravidae (n = 24)	7	9	11^a^	2	2	5
**Sequences**	Primigravidae (n = 33)	11	20^a^	1	7^a^	8^a^	0
	Multigravidae (n = 37)	11	14	12	2	2	5^a^

Gap, EDTKQ and EYTGN motifs are mainly found in samples from primigravidae compared to those from multigravidae (p = 0.02) whereas TFKNI and QYTGN are mostly or exclusively found in multigravidae (p = 0.013). At the level of sequence types obtained from each sample, the EDTKQ, EYTGN and gap signatures are more frequent among DBL5ε sequences from primigravidae compared to those originating from multigravidae (p = 0.036). Similarly, the TFKNI and QYTGN motifs are mostly or exclusively found in sequences from multigravidae (p = 0.0019 and p = 0.038 respectively).

a p<.05, Fisher's exact test.

b p<.001, Fisher's exact test.

### Expression of distinct variants of recombinant VAR2CSA DBL5ε from placental parasites

**Figure 4:**
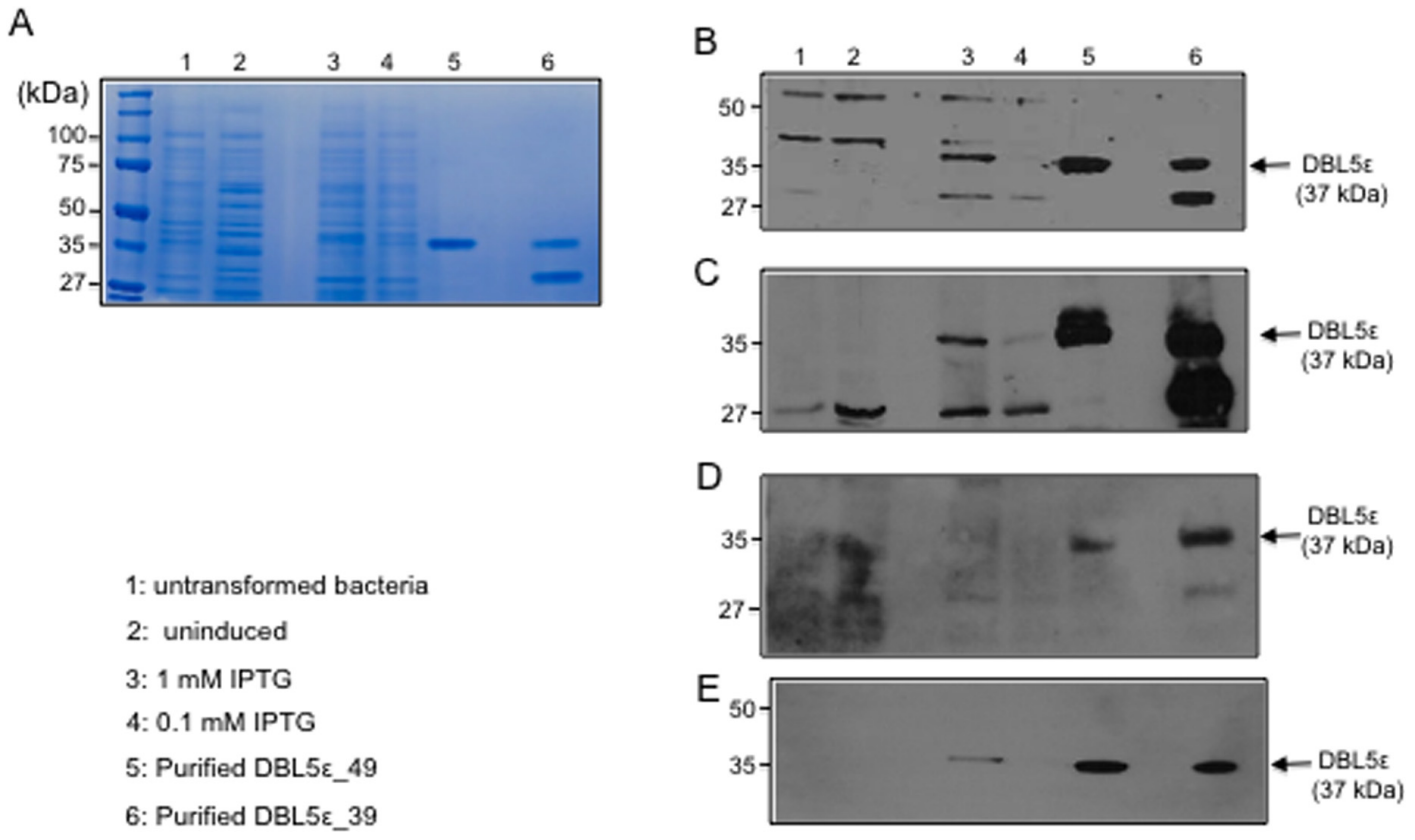
Bacterial recombinant DBL5ε domain of VAR2CSA expression. Lysates of untransformed (lane 1) bacteria, DBL5ε_CYK49 [uninduced (lane 2), induced 1mM IPTG (lane 3), induced 0.1 mM IPTG (lane 4)], DBL5ε_CYK49 (lane 5) and DBL5ε_CYK39 (lane 6) after two purification steps were subjected to SDS/PAGE and either stained with Coomassie blue (A) or immunoblotted with either purified IgG multigravidae plasma (B), antisera from mice vaccinated with DBL5ε_CYK39 (C), antisera from DBL5ε_CYK49 vaccinated mice (D) or monoclonal anti-histidine antibodies (E). 30 µg of bacteria-expressed-extract proteins and 2 µg of purified domains were used for analysis. Immune complexes were detected with appropriate horseradish peroxidase coupled antibodies.

Two VAR2CSA DBL5ε variants (CYK39 and CYK49) were produced in *Rosetta gami* DE3 strains. Both variants were chosen for analysis as *P. falciparum* IEs corresponding to isolate CYK39 have been described as high CSPG binders and parasites from CYK49 as low binders [2]. The *Rosetta gami* bacteria strain allows the formation of disulfide bonds that could favour production of biologically active proteins. Protein production was induced with 0.1 and 1 mM IPTG. The soluble protein produced was affinity-purified, subjected to gel filtration, and the purity was checked by SDS-PAGE (Figure 4A) and Western blotting. An average of 5 mg of pure protein was obtained after the different purification steps. Western blot analysis showed that total IgG purified from a plasma pool of malaria exposed multigravidae labelled a single dominant band of 37 kDa in 1 mM IPTG induced bacterial extract and in purified DBL5ε (Figure 4B). The same product (37 kDa) was identified by specific IgG generated in mice by DNA vaccination with DBL5ε_CYK39 (Figure 4C) and DBL5ε_ CYK49 IgG (Figure 4D), as well as with anti-histidine tag monoclonal antibodies (Figure 4E). Bands of expected size were observed neither in the untransformed nor uninduced bacterial extracts (Figure 4).

### *In vitro* binding ability of placental parasite recombinant DBL5ε VAR2CSA variants to CSPG

**Figure 5:**
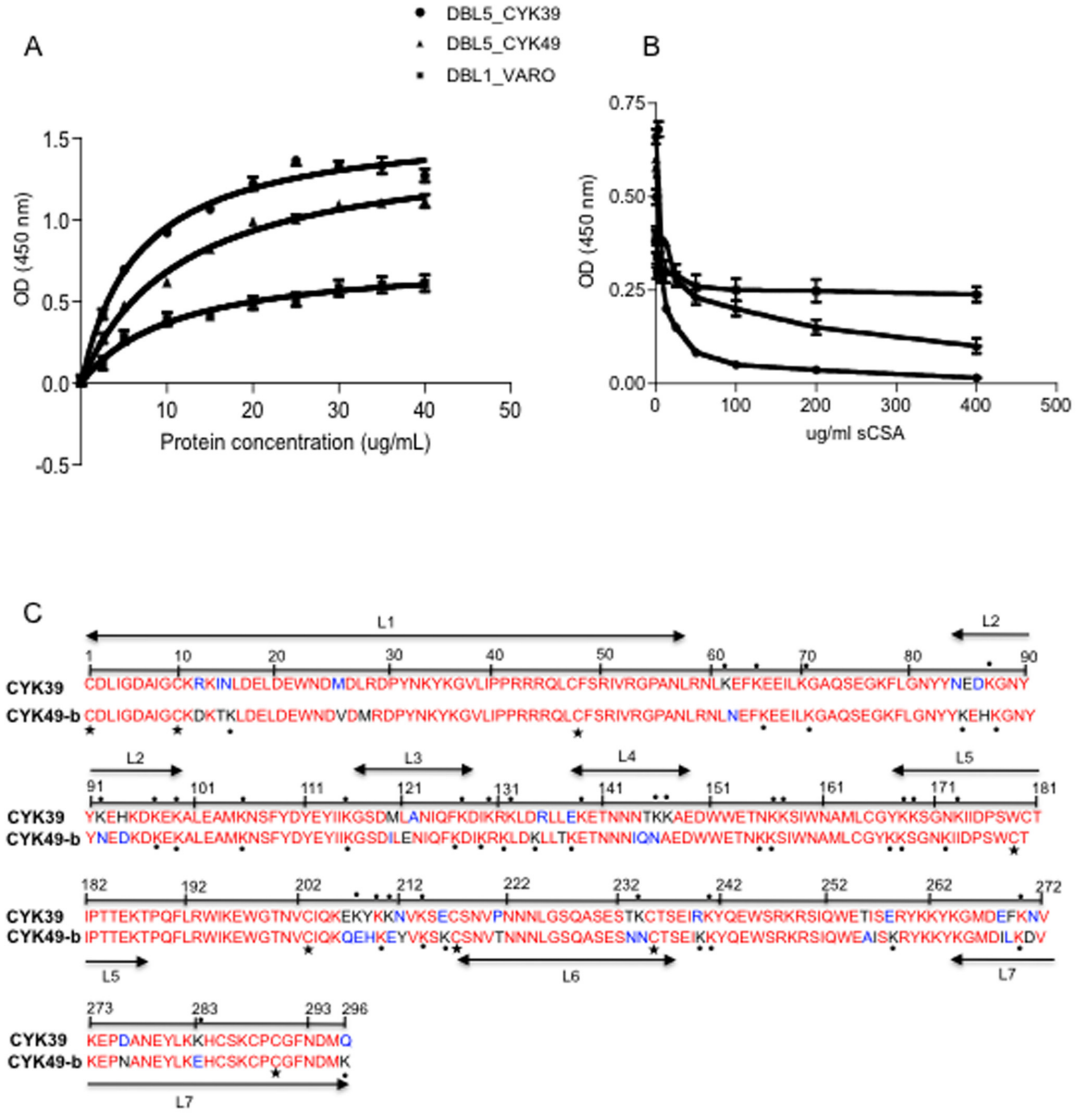
CSPG binding of the DBL5ε domain of the VAR2CSA from parasite isolates. (A): Increasing concentrations of protein were added to wells coated with 5 µg/ml of CSPG. CSPG-binding of the DBL5ε_CYK39 (circle), DBL5ε_CYK49 (triangle) and the non CSA-binding VARO NTS-DBL1α domain used as control (square). Results are the means of three binding assays and the error bars indicate the standard deviation. (B) Inhibition assay. Recombinant DBL5ε variants (5 µg/ml) were pre-mixed with increasing amounts of soluble CSA 0.25–400 µg/ml, and binding to CSPG-coated plates was determined. Results are the means of three inhibition binding assays and error bars indicate the standard deviation. (C): Sequence comparison of VAR2CSA DBL5ε domains from CYK39 and CYK49. Asterisks and circles indicate respectively Cystein residues and Lysine. Conserved amino acids are shown in red and polymorphic residues in black. The 7 loops (L1–L7) identified according to Andersen P *et al.* [8] are underlined.

The CSPG binding capacity of the two DBL5ε variants was estimated by ELISA. Both variants showed a relatively higher binding ability to CSPG compared to NTS-DBL1α domain of VARO (Figure 5A). This interaction was concentration-dependent. In this model, the NTS-DBL1α domain of VARO also produced in *Rosetta gami* displayed weak binding ability to CSPG. To determine whether this interaction was CSPG-specific, we tested the ability of soluble CSPG (decorin) or CSA (bovine trachea CSA) to compete for protein binding on a CSPG pre-coated plate. As shown in Figure 5B, soluble CSPG like soluble CSA (data not shown) indeed competed for binding observed on CSPG. Sequence comparison of both DBL5ε variants expressed showed that they were highly similar but contained 31 different residues. Moreover, positively charged amino acids appeared to be differentially expressed in both variants (Figure 5C). As position 303 seemed to be associated with binding density, the sequences were analysed for mean CSA and CSPG binding densities and the difference associated with the occurrence of the Q, E and K residues. Indeed, high CSA or CSPG binding affinity was mainly associated with residue Q_303_ (p = 0.005) whereas low CSA or CSPG binding affinity was associated with E/K_303_ (Table 2). Interestingly as shown in figure 5C, the equivalent residue for CYK39 and CYK49 sequences was in fact Q_296 and_ K_296_ respectively. The mapping of Q_303_ on structural model indicates that this residue seems to be surface exposed, but located in the bottom of what could be a binding pocket (Figure 2D).

**Table 2:** VAR2CSA-DBL5ε residues Q_303_, E_303_ and K_303_ distribution in relation to placental parasite CSA/CSPG binding affinity.

	Q_303_	E_303_	K_303_
**Isolates**			
**High binders (n = 20)**	10/20^a^	6/20	6/20
**Low binders (n = 16)**	1/16	7/16	9/16

Those parasites ability to bind CSA or CSPG have previously been described [2].

a p<.01, t-test.

n corresponds to placental parasite isolates.

### Antibodies against DBL5ε domain of VAR2CSA increase in a parity-dependent manner

**Figure 6:**
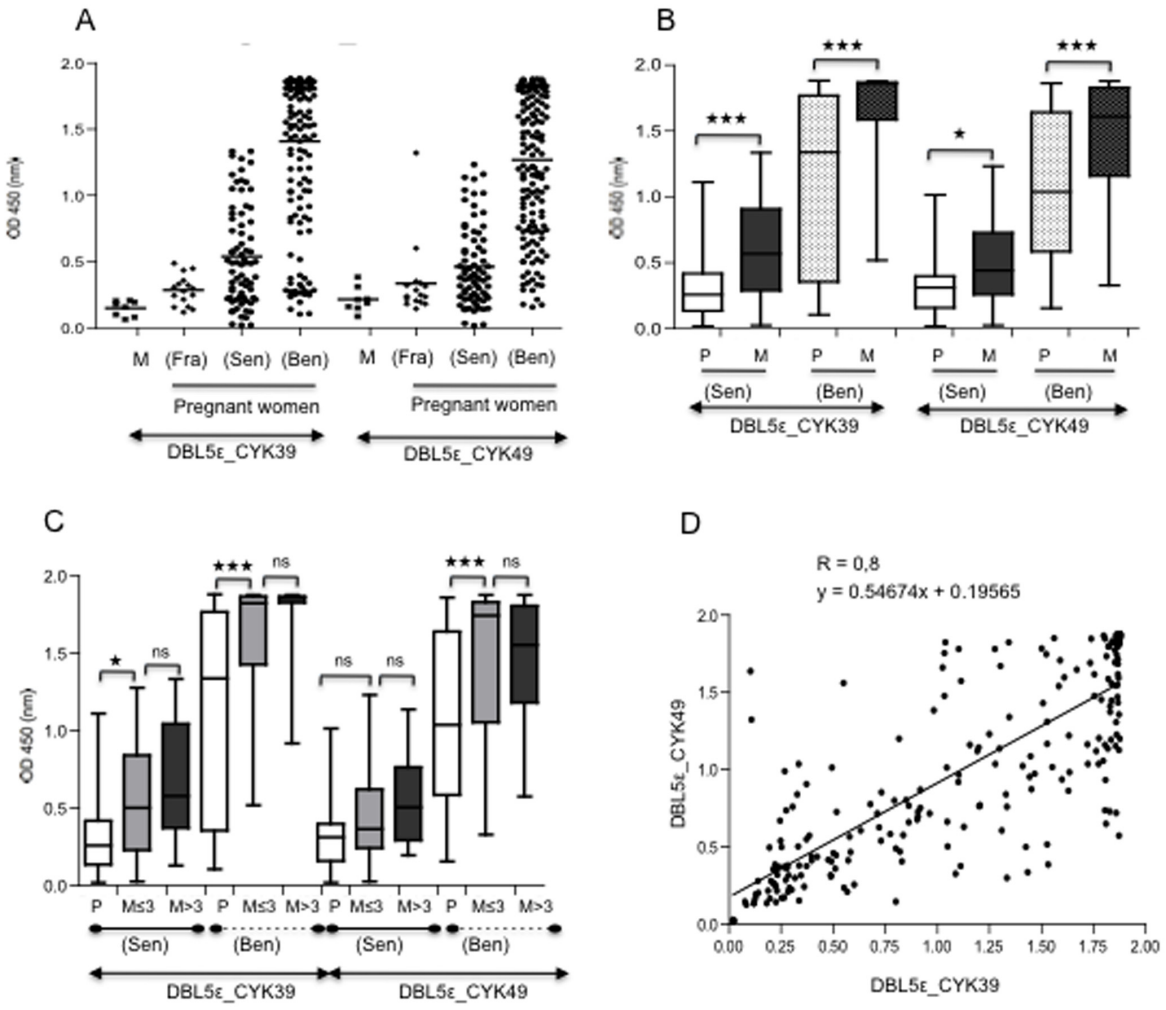
Plasma reactivity against DBL5ε domains of VAR2CSA. (A): Plasma levels of IgG with specificity for DBL5ε domain of VAR2CSA in 8 French unexposed men (M), 16 French unexposed pregnant women (Fra), 75 Senegalese pregnant women (Sen) and 160 Beninese pregnant women (Ben). DBL5ε variants CYK39 and CYK49 were tested. (B): Plasma levels of VAR2CSA DBL5ε domain according to parity. DBL5ε antibodies levels were quantified in the same groups of malaria-exposed pregnant women (Benin and Senegal) as in A. 24 primigravidae (P), 51 multigravidae (M) from Senegal; 80 primigravidae and 80 multigravidae from Benin. (C): Plasma levels of VAR2CSA DBL5ε domain according to parity range. Malaria exposed women used in (B) were separated in three groups; primigravidae (P), women whose parity level is lower or equal to 3 (M≤3) [Beninese women: n = 48, n = 26 for Senegalese women] and those whose parity status is higher than 3 (M>3) [Beninese women: n = 32, n = 25 for Senegalese women]. (D) Correlation between the reactivity to each DBL5ε variant in a given plasma.

Recombinant DBL5ε variants (CYK39 and CYK49) were used to assess the plasma levels of anti-VAR2CSA IgG. Independent of which variant was tested, antibodies with specificity for *Rosetta gami*-produced DBL5ε VAR2CSA were seen only in plasma from *P. falciparum*-exposed pregnant women living either in Benin (Ben) or in Senegal (Sen) (Figure 6A). In contrast, plasma levels of antibodies against the recombinant DBL5ε were insignificant in both French unexposed men (M) and pregnant women (Fra) (Figure 6A). Detailed analysis of *P. falciparum*-exposed pregnant women indicated that for each antigen tested, Senegal and Benin multigravidae (M) had significantly higher levels of DBL5ε antibodies than primigravidae (P); (CYK39: both p<0.0001; CYK49: p = 0.019 for Senegalese and p<0.0001 for Beninese; Figure 6B), however contrary to Senegalese primigravidae, most Beninese primigravidae presented with high DBL5ε VAR2CSA antibody levels (Figure 6B). A fine analysis of the plasma reactivity of the women demonstrates that antibodies against DBL5ε increased with parity (Figure 6C). We compared plasma levels of VAR2CSA specific IgG using both DBL5ε recombinant proteins. Cut-off values were set to mean + 2SD (plus two standard deviations) of reading obtained with the negative control plasma samples. The percentage of antibody reactivity considered to be positive was 80% for DBL5ε_CYK39 and 60% for DBL5ε_CYK49. Despite a homology of 80%, there is a significant difference of reactivity between both variants (Chi^2^ test p = 0.005). A comparative study of the reactivity of each plasma with respect to each of the variants shows that the response to both variants was strongly correlated (Pearson's test r = 0.8, p<.0001; Figure 6D), confirming that the VAR2CSA DBL5ε domain contained conserved epitopes.

### Evidence of conserved cross-reactive epitopes in DBL5ε VAR2CSA

**Figure 7:**
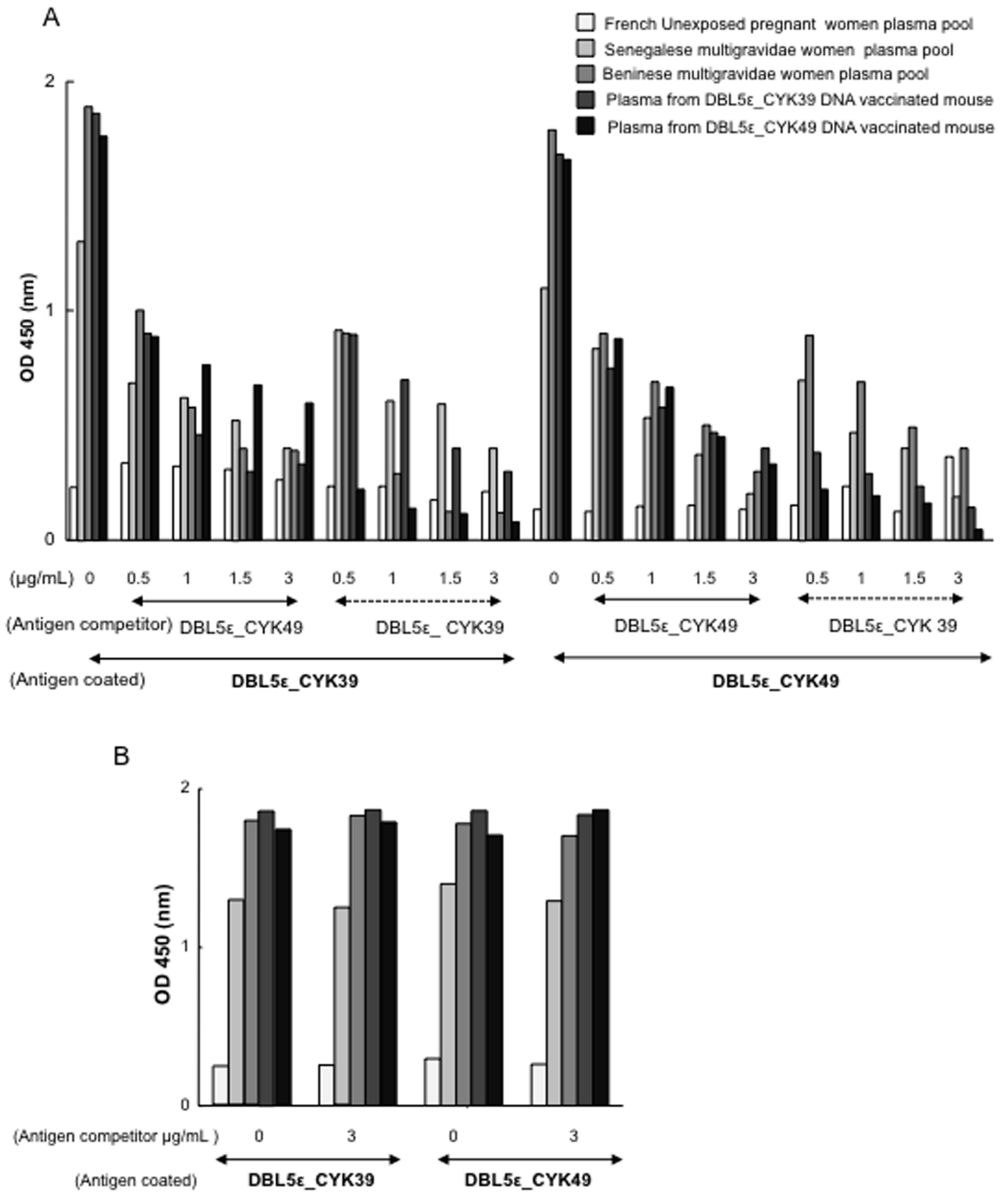
Cross-reactive antibody target between VAR2CSA DBL5ε variants. Cross-reactivity was determined by competition ELISA using either a multigravid plasma pool with high titer of VAR2CSA-specific antibodies (Beninese or Senegalese women), plasma from DBL5ε_CYK39 or CYK49 DNA genetic vaccinated mouse (A). NTS-DBL1α domain of VARO was used as negative control (B). Each colour shows the reactivity with the indicated antibodies.

**Figure 8:**
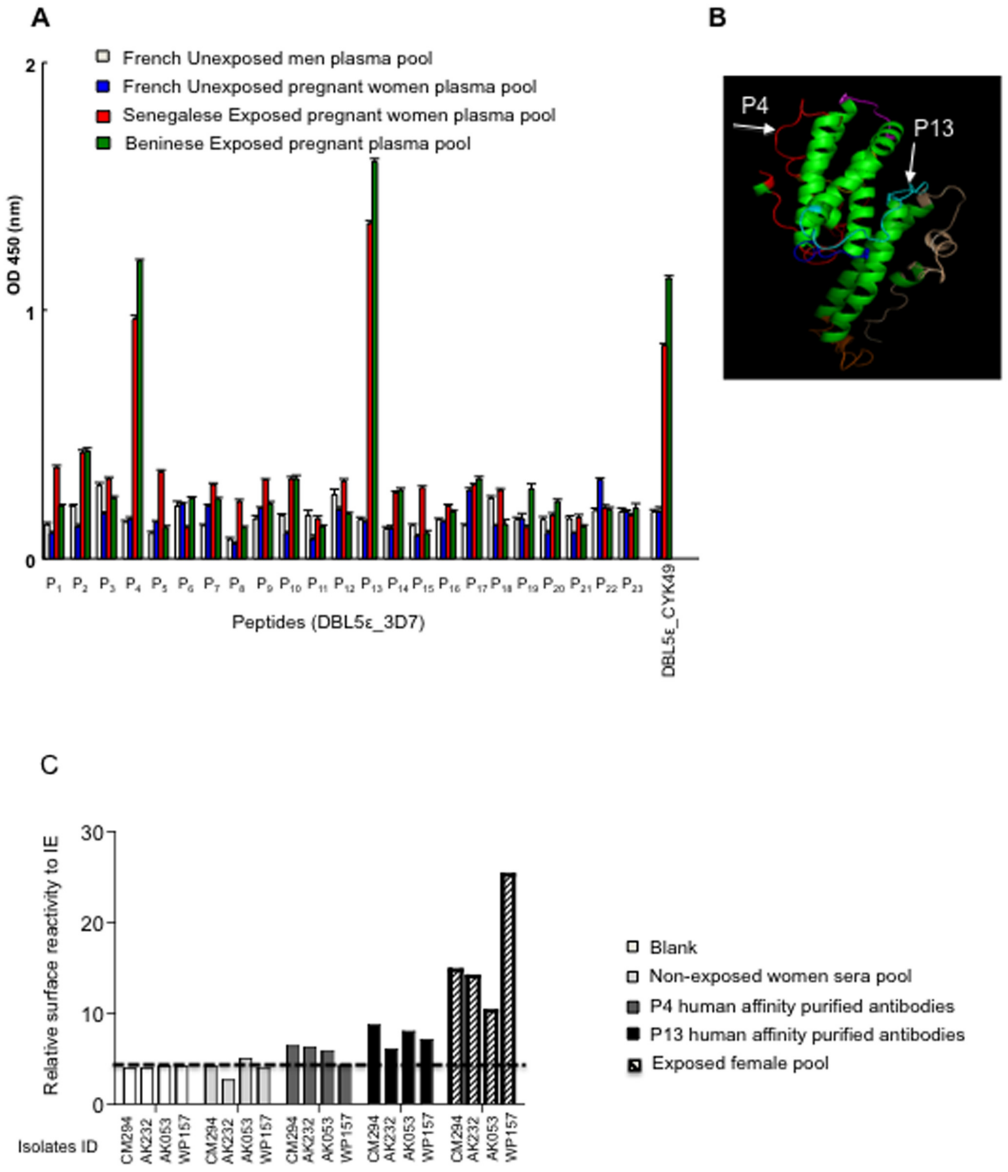
Reactivity of human specific conserved DBL5ε affinity purified antibodies with *P. falciparum* infected erythrocytes. (A): IgG recognition of 3D7-DBL5ε peptides library. (B): Mapping of P4 and P13 peptides on DBL5ε model [8]. (C): Senegalese women antibodies were affinity purified on peptides P4 and P13 and tested for reactivity against PAM Beninese parasite isolates. Flow cytometry analysis of human affinity-purified IgG against peptides P4 and P13 against PAM parasite isolates. Each colour shows the reactivity to native parasites with the indicated antibodies. Four isolates were tested with each IgG. Sample without primary antibody (blank), non-exposed women plasma pool, and exposed women plasma pool are used as control respectively.

Recombinant DBL5ε variants were used in competition ELISAs to demonstrate that DBL5ε domain of VAR2CSA contains cross-reactive epitopes. While one variant of the two expressed VAR2CSA DBL5ε was used for coating, the other one was used as soluble competitor. The antibody reactivity of either a high-titered VAR2CSA plasma pool from Beninese or Senegalese women, or antisera to DBL5ε_CYK39 and DBL5ε_CYK49 generated in mice by DNA vaccination, or plasma pool from unexposed French pregnant women was compared with or without pre-incubation with increasing concentrations of the competing VAR2CSA DBL5ε variant. As negative control, all plasma were incubated with VARO NTS-DBL1α domain. Figure 7 shows that DBL5ε from placental parasites contains conserved epitopes. Indeed, whichever the DBL5ε variant tested, the competitor inhibited its antibody recognition in a concentration-dependant manner (Figure 7A). No significant competition was seen with the negative VARO control protein (Figure 7B). Due to the highly conserved nature of VAR2CSA DBL5ε sequence, it was decided to determine whether any of its conserved regions was recognised by naturally acquired antibodies. We synthesised a library of peptides using 3D7 DBL5ε sequence. All peptides were screened in ELISA for reactivity against a plasma pool from Beninese or Senegalese women, French unexposed pregnant women and men. Two peptides P4 and P13 located in highly conserved regions of VAR2CSA displayed significant and specific recognition by plasma of malaria exposed pregnant women compared to control plasma from French unexposed pregnant women and men (Kruskal-Wallis test, p<0.0001; Figure 8A). Antibody reactivity of both peptides was higher in multigravidae compared to primigravidae, though not significant (Mann-Whitney U, p = 0.17).

### Specific antibodies to VAR2CSA DBL5ε conserved peptides mark native VAR2CSA on the surface of infected erythrocyte

Mapping of both peptides P4 and P13 on DBL5ε structural model indicated that both of them are surface-exposed (Figure 8B). Furthermore, specific antibodies against both peptides were affinity-purified from the Senegalese pregnant women plasma pool and allowed to react with PAM parasites collected from pregnant women from Benin. The pregnancy specific antibody recognition of the isolates used was checked prior by FACS with human plasma control pools (data not shown). The results presented on Figure 8C show that the antibodies with specificity to the selected peptides reacted with the native VAR2CSA expressed by PAM parasites on the surface of IE.

## Discussion

Pregnant women acquire protective antibodies that cross-react with geographically diverse placental *P. falciparum* isolates, suggesting that surface molecules expressed on infected erythrocytes (IE) by PAM parasites have conserved epitopes and, thus, that a PAM vaccine may be possible to achieve. The search for surface antigens of placental *P. falciparum* parasites is focused on the PfEMP1 family. Most studies in recent years have shown that VAR2CSA is the dominant PfEMP1 associated with parasite binding to the placenta. Due to technological difficulties the exact conformation of the entire VAR2CSA protein remains unknown. Preliminary studies to understand its binding properties focused on its DBLs domains and functional studies have shown that several VAR2CSA DBLs including DBL5ε can individually bind CSA *in vitro*. This approach has become questionable as no efficient anti-adhesion antibodies for IE have been obtained following vaccination with a single domain. Recent studies have nevertheless demonstrated that VAR2CSA DBL5ε domain can induce antibodies with a broad range of reactivity against placental isolates [16], [20] and therefore may represent a potential target for PAM vaccine development. This study analysed sequence variation in the DBL5ε domain of the transcribed *var2csa* gene from multiple placental parasite isolates. The aim was to evaluate antigenic diversity and diversifying pressure within this attractive VAR2CSA area. Using cDNA (complementary acid deoxyribonucleic) from 40 placental parasite isolates from a previous study, the region encoding DBL5ε+Id5 of *var2csa* was amplified, cloned and sequenced. Findings from our study population clearly confirmed previous observations that the VAR2CSA DBL5ε is highly conserved [18]. Indeed, an average of 81% amino-acid sequence identity was seen among DBL5ε sequences as reported by Guitard et al. on a different study population [18]. Variations were mainly located in segments of variable length and mapping of DBL5ε regions to 3-D model revealed that variable areas are located in the loops and protruding helices [8].

Two variable regions, one in the DBL5ε and another one in the Id5 sequences appeared to be of particular interest regarding the bias in motif distribution among gravid women. Three significant motifs (gap, VFNNA and TFKNI) were identified in the first region spanning Aa from position 275 to 279. Despite the relatively high variability of the Id5, another area with motif segregation (EDTKQ, EYTGN and QYTGN) was found between Aa 303 and 313. The major observation in these sites is the significant difference between motif occurrence among parasites from primigravidae and multigravidae. Certain motifs are preferentially found in parasites from primigravidae (gap_275–279_, E_303_D_308_T_309_K_312_Q_313_ and E_303_Y_308_T_309_G_312_N_313_), whereas others are only found in parasites infecting multigravidae (TFKNI_275–279_ and QYTGN_303–313_). Interestingly, most of the parasites with QYTGN_303–313_ motif also had TFKNI_275–279_. Those expressing either EDTKQ_303–313_ or EYTGN_303–313_ are mostly associated with a gap_275–279_. Such selection pattern was already seen in the DBL3X sequence and plausible explanations can be given, based on several hypotheses: (i) either that parasites infecting primigravidae are the most efficient mediators for binding and therefore have a biological advantage in women with limited immunity against PAM, (ii) or that the parasite variants mostly found in primigravidae are the more common in the area and therefore are more likely to infect exposed primigravidae while multigravid women already have developed specific antibodies during previous pregnancies. The tropism of certain parasite variants for multigravid women suggest that some rarer variants, probably not the most virulent can escape existing immunity to common VAR2CSA variants. These findings have important implications for understanding immunity to PAM in a context where the development of a VAR2CSA-based vaccine is gaining interest. Further analyses in this study also found a significant difference at a site situated in the Id5 according to the ability of IE to bind CSA or CSPG *in vitro*. Isolates with high binding affinities associated with Q_303_ and low CSA/CSPG binders associated with E/K_303_. This could indicate that conservation of Q_303_ may have conformational importance for maintaining high binding ability by the IE.

The results generated in the present study highlight the fact that fundamental gaps remain in our knowledge and understanding of placental parasites. Protection against PAM is consistent with repeated exposure during pregnancy to previously unknown antigens. Most of multigravidae infected by parasites with the TFKNI or QYTGN motifs have a parity status above 3, suggesting that despite the protection acquired during different pregnancies, women can still be infected by new parasite variants [18]. In the context of developing an optimal VAR2CSA-based vaccine that can protect against placental malaria, it will be particularly useful to overcome the challenges associated to sequence variation in this interesting candidate. The relation underlying the even limited variations described in this study suggests that these can have critical implication in the functionality of the whole molecule including its ability to subvert immunity. Our results clearly indicate that the design of a protective vaccine based on VAR2CSA should not be limited to a single variant. A limited number of variants may be sufficient for broad coverage, provided sites under significant variations are considered.

We have characterized two distinct variants of DBL5ε from our study population. The measure of plasma levels of the antibodies against these two DBL5ε variants showed that the two proteins were broadly recognized by samples from two malaria endemic regions with different *P. falciparum* transmission levels. Both VAR2CSA DBL5ε variants were recognized in a parity-dependent manner although the acquisition of immunity against VAR2CSA differed between the two regions. In areas of intense *P. falciparum* transmission, pregnant women generally develop protective immunity to PAM over successive pregnancies, and only primigravidae and secundigravidae present higher placental infection prevalence rates [24]. In *P. falciparum* transmission areas such as Benin, exposure is high and results in a fast acquisition of immunity while the acquisition may be delayed in areas of low and seasonal transmission such as in Senegal. In our two populations, multigravidae presented with higher antibody levels against VAR2CSA than primigravidae; but in Benin, where transmission is perennial, the mean antibody level was overall higher than that of women from Senegal. Among primigravidae, 57% of Beninese had anti-VAR2CSA antibodies at delivery compared to 16% of Senegalese. This could be explained by difference in malaria exposure in the study areas. A close comparison of the two VAR2CSA DBL5ε recombinant variants demonstrated that, despite a homology of 80% in their amino acid sequences, both variants presented some distinguishable characteristics. The DBL5ε_CYK39 exhibited a higher CSPG binding ability and a higher recognition by plasmas than the DBL5ε_CYK49 variant, although both constructs showed parity-dependent recognition patterns. This observation suggests that some variants can be more readily recognized than others. This can also be a useful consideration in vaccine development strategy, as not all VAR2CSA variants are likely to yield broad and high recognition or reactivity.

In the variable regions of DBL5ε distinct motifs were identified, the sero-reactivity of peptide containing TFKNI (P19) was assessed by ELISA. No reactivity was observed against this as shown in Figure 8A. Nevertheless, this result is not surprising as we clearly showed that TFKNI only were encountered in women presenting high parity status and may be expressed by uncommon variants. In the same effort to develop optimal VAR2CSA-based vaccine, it is advisable to target highly conserved residues or as many residues as possible that are accessible by host immune response to broaden the possibility of reaching all potential parasite populations expressing the VAR2CSA ligand. From the current observation it is obvious that like DBL3X, the DBL5ε domain variants share common and cross-reactive motifs. We identified two peptides (P4 and P13) in the highly conserved region of the DBL5ε domain that significantly reacted with plasma pool from pregnant women of different endemic areas. Affinity-purified antibodies against those peptides specifically reacted with placental parasites, confirming that these peptides are actually surface-exposed, as suggested by the 3D model. One such epitope in DBL5ε (peptide P63) was previously described which reacted strongly with Tanzanian female plasma [8]. DBL5ε peptide P4 identified in this study has 16 amino acids out of 20 in common with P63 peptide. Existence of such conserved and accessible epitopes supports the broad recognition observed on this particular DBL domain and emphasizes on its potential interest.

Knock-out studies have previously demonstrated the exclusive need for VAR2CSA to mediate IE binding to CSA [11], and it has been shown that four of the six Duffy-binding-like (DBL) domains of VAR2CSA individually have the ability to bind CSA *in vitro* [12], [13], [14], [15], [16], In this study, we confirmed the CSA-binding ability of recombinant DBL5ε to CSPG. Our results have demonstrated in our experimental conditions, that both placental isolate DBL5ε variants have certain affinity for CSPG. This result is in agreement with the fact that DBL5ε _CYK39 variant is able to bind to CSA and heparin sulfate [16]. However NTS-DBL1α domain of the VARO PfEMP1 that is not involved in the placental sequestration of parasites also presented a weak affinity to CSPG. The binding of VAR2CSA to placental CSPG plays a major role in malaria during pregnancy, and the understanding of this interaction will be valuable to define easily producible constructs that can induce adhesion inhibitory antibodies. Unlike CSA binding that is unique to PAM parasites, *in vitro* interaction of individual DBL with CSA is often seen with non-VAR2CSA DBLs. Whether such interactions of individual domain can predict for IE binding phenotype is debatable. Thus the CSPG interaction was used in the current study only as an analytic tool to characterize the properties of the both recombinant DBL5ε variants expressed. Recent studies have demonstrated that even though DBL3X and DBL6ε can bind to the same ligand, the sites of interaction differ in these domains [25], [26]. Nevertheless, in each of these domains, the binding site involves residues that are conserved in parasite isolates from different geographic locations. We report in this study a difference in CSPG binding ability among two VAR2CSA DBL5ε variants. The structure of this domain has not yet been solved and residues which are essential for interaction are not identified.

In summary, we demonstrated for the first time that although VAR2CSA DBL5ε sequence has a limited antigenic diversity, it contains some molecular signatures that distinguish parasites according to the host parity. These findings have important implications for vaccine design based on VAR2CSA. Malaria-exposed women also develop antibodies against conserved parts of VAR2CSA DBL5ε domain. Two of such conserved epitopes were identified here and, naturally acquired antibodies to them stained native proteins on placental parasites. Our data support the importance of DBL5ε in the current effort of elucidating the parts of the VAR2CSA protein that can induce an antibody response with broad reactivity on placental parasites.

## Materials and Methods

### Parasite isolates

All *P. falciparum* PAM parasites for which sequences were generated were collected at delivery in a cross-sectional study conducted in Senegal in 2003[2]. Samples from 39 *P. falciparum* isolates were available for the study. The mean ± SD age of women who donated the parasites was 24±6.5 years. They were composed of 15 primigravidae, 6 secundigravidae, and 18 multigravidae. *P. falciparum* infected erythrocytes (IEs) were collected from parasitized placentas (parasite density ranging from 0.1% to 50%; mean ± SD,12.8±12.7) by flushing as previously described [2]. Collected IEs were conserved in Trizol LS (Invitrogen) and stored at −80°C until use. The binding ability of parasite isolates to CSA were evaluated [2]. Neonate birth weight was estimated by use of an electronic balance. There were 56% low birth weight LBW (<2500g) recorded.

Placental parasite “748” was collected in Tanzania, as described elsewhere [20].

Parasites used to evaluate antibody reactivity with the surface of IEs were freshly collected from pregnant women enrolled in the on going STOPPAM project based in the district of Comè, southwestern Benin [27].

### Plasma samples collection

Plasma samples from malaria exposed women are from two different malaria endemic areas: Perennial (Benin) and seasonal (Senegal) *P. falciparum* transmission. Senegalese pregnant women were enrolled in a cohort study in 2001 in Thiadiaye [7]. Women presenting with fever and a positive blood smear were given curative treatment with chloroquine, the drug advocated in Senegal at the time of study for both prophylaxis and treatment.

In Benin, as described [28], pregnant women were enrolled in a cohort study conducted from July 2005 through April 2008 in Ouidah, a semirural town in Benin that is located 40 km west of Cotonou, the political capital of Benin. Perennial malaria transmission with seasonal peaks is mostly attributable to *P. falciparum* [29]. Sulfadoxine-pyrimethamine or mefloquine was given to women during the study.

Plasma samples from 24 French pregnant women and 8 adults men without *P. falciparum* exposure were used as negative controls.

All women plasma samples tested in this study were collected at delivery time.

### Cloning and sequencing of placental var2csa DBL5ε genes

All VAR2CSA DBL5ε sequences were obtained from placental parasites complementary DNA (cDNA). Total RNA was extracted from parasites conserved in Trizol according to the manufacturer's instruction. The total RNA concentration was determined at 260 nm and RNA integrity was checked in 1% agarose gel. RNA samples were pretreated with DNAse I (Sigma-Aldrich). 5 U of RNase-free DNase per 5 ug of RNA was incubated at 37°C for 30 min, followed by 10 min heat inactivation at 65°C. All RNA samples were subsequently tested in real-time PCR for contamination with genomic DNA using a primer set for the housekeeping gene *seryl-tRNA synthetase*. cDNA was synthetised by reverse trancriptase (Superscript II, Invitrogen) and random hexamer primers, as described by the manufacturer. All VAR2CSA DBL5ε sequences were amplified using high fidelity enzyme (Phusion) with the following universal primers designed in highly conserved areas flanking the DBL5 DBL5ε+the hypervariable interdomain (Id5): DBL5ε Forward: 5′-GTC ACC CCC GGG GAC AAT GCA ATA AAA GAT TAC and DBL5ε Reverse: 5′-TAG GCA TTT GCG GCC GCC TTC AAG TTC AGC TGG AAT ATT. Two µl of cDNA was used for the PCR reactions. PCR products were inserted into a pAcGP67C Baculovirus Transfer Vector (BD). Ten to 15 colonies of each cloning were sequenced by GATC (www.gatc.com).

### Cloning, expression and purification of recombinant VAR2CSA DBL5ε variants proteins

DBL5ε sequence from placental parasite isolate CYK 49 [2] was amplified from the corresponding cDNA with the following primers: 5′ ACT GGC AGG AAT TCA TGT TTG ATG ATC AGA CA and 3′ ATC GAC TGG CAG GCG GCC GCT TAA TGG TGA TGG TGA TGG TGT TTC ATA TCA TTA. PCR product was digested with *Eco*R1 and *Not*1 for cloning into the modified bacterial expression vector pET-21 (Novagen, http://www.novagen.com) to produce His-tagged recombinant proteins in *Rosetta gami* strain. The ligated vectors were transformed into *E. coli* DH5α strain, and positive clones were selected with ampicillin resistance. *Rosetta gami* cells transformed with recombinant plasmids, were cultured into LB broth containing ampicillin (50 µg/ml) at 30°C, and treated at the mid-log phase (OD_600_ = 0.4) with IPTG, to induce protein production. Cells were cultured at 25°C overnight, and harvested by centrifugation at 6,000 g at 4°C for 15 min. The pellet was washed, resuspended in cold buffer containing 10 mM Tris, 500 mM NaCl and protease inhibitor cocktail (Cocktail set N°III, Calbiochem), and sonicated. DBL5ε recombinant protein was purified from bacterial soluble fraction on Ni^2+^ metal-chelate agarose columns (GE Healthcare), and eluted with 10 mM Tris, 500 mM NaCl and 150 mM imidazole. Affinity chromatography step was followed by gel filtration. Recombinant DBL5ε protein from isolate CYK *39* [16] and NTS-DBL1α VARO [30], [31] were produced, and purified under the same conditions.

### Antibodies production

Specific antibodies to DBL5ε CYK39 or DBL5ε CYK49 were induced in mice by genetic immunization. Briefly, DNA injections were subcutaneously electro-transferred to 6-week-old Swiss female mice (Janvier, France) using 40 µg of plasmid DNA encoding either DBL5ε_CYK39 or DBL5ε_CYK49. All plasmids used for genetic vaccination are based on a pVax1 vector back-bone (Invitrogen) in which the original cytomegalovirus (CMV) promoter has been replaced with the CMV promoter of the pCMVb plasmid (Clontech), as described [32]. Mice were electro-transferred on days 0, 21 and 45. Mice were bled before each electroporation, and a full bleed was collected 80 days (D80) after the first electroporation. Immune response was checked by ELISA on consecutive bleeds. All procedures complied with European and National regulations.

IgG from plasma of multigravidae living in an endemic area were purified on a Hi-Trap protein G HP column according to the manufacturer's recommendations (GE-Healthcare). The specificity of the purified antibodies was tested in ELISA against recombinant DBL5ε recombinant proteins (CYK39 and CYK49).

### VAR2CSA proteins characterization by Western blotting

The soluble recombinant VAR2CSA DBL5ε proteins were checked by Sodium Dodecyl Sulfate-polyacrylamide gel electrophoresis and Western blotting. Protein samples (2–50 µg) were suspended in Laemmli-buffer (Tris/HCl 62.5mM, pH6.8, 2% SDS, 5% β-mercaptoethanol and 10% glycerol), subjected to SDS-PAGE [33] using a 4–12% acrylamide slab minigel (Invitrogen, Carisbad, CA, USA). Western blotting was performed with (2–30 µg) bacterial (induced, uninduced and nontransformed) lysates or purified eluates electrophoresed through 4–12% SDS-PAGE gels and electro-transferred to 0.2 µm Protan BA 83 nitrocellulose sheets (Schleicher & Schuell) for immunodetection. The membranes were blocked for 1 h with 5% nonfat dry milk in phosphate-buffered saline (PBS) with 0.1% Tween® and then incubated separately with either a 1∶5000 dilution of a monoclonal anti-histidine HRP conjugated antibody (46-0707, Invitrogen) or a 1∶1000 dilution of DBL5ε_CYK39 or DBL5ε_CYK49 antiserum from vaccinated mouse D50 (day 50) or 1∶1000 of IgG purified from plasma of multigravidae living in an endemic area. Immune complexes were detected with a HRP coupled with either anti-mouse IgG antibody (1∶10 000, AP127P Sigma-Aldrich) or anti-human IgG antibody (1∶10 000, A0170 Sigma-Aldrich).

### Competition ELISA, peptide ELISA and affinity purification of antibodies

Prior to competition ELISA, both VAR2CSA DBL5ε constructs were used to assess the plasma levels of anti VAR2CSA IgG of 160 malaria exposed pregnant women from Benin (primigravidae n = 80, multigravidae n = 80) and Senegal (primigravidae n = 24; multigravidae n = 50), French unexposed pregnant women (n = 16), and French unexposed men (n = 8). ELISA was carried out on plates coated with 0.5 µg/ml of the DBL5ε. The IgG plasma levels were expressed as Optical densities (OD) values read at 450nm. A pool of plasma samples from unexposed French pregnant women was used as a negative control whereas a pool of plasma samples from multigravidae pregnant Senegalese women, previously demonstrated to have high levels of anti-VSA IgG (VSA: Variant surface antigen) against placental isolates, was used as a positive control.

For competition ELISA, microtiter plates (Nunc 442404) were coated with each antigen (DBL5ε_CYK39, DBL5ε_CYK49, NTS-DBL1α-VARO, 0.5 µg/ml in PBS). Five different plasma pools were individually pre-incubated for 2 h at room temperature (RT) with increasing concentrations of competing antigen (0.5, 1, 1.5, and 3 µg/ml): Beninese pregnant women plasma pool (diluted 1∶500), Senegalese pregnant women plasma pool (diluted 1∶500), DBL5ε_CYK39 plasma from DNA vaccinated D50 (1∶100 000), DBL5ε_CYK49 plasma from DNA vaccinated D50 (1∶40 000), and French unexposed women plasma pool (1∶100). After incubating the plates with blocking buffer (PBS, 0.5 M NaCl, 1% Triton X-100, 1% BSA) for 2 h at RT, the pre-absorbed pool were added to the antigen-coated wells in duplicate and incubated overnight at 4°C. In addition to the pre-absorbed plasma pool, a non-absorbed pool was included for each coating antigen. Following washing of the plates four times with washing buffer (PBS, 0.5 M NaCl, 1% Triton X-100, pH 7.4), the secondary antibody (Goat anti-human IgG HRP, A0170, Sigma-Aldrich for human plasma and Goat anti-mouse IgG HRP, AP127P, Chemicon) diluted 1∶4000 in blocking buffer was added, and incubated for 1 h at RT. Plates were washed four times, and antibody reactivity visualized by the addition of TMB (Tetramethylbenzidine). Coloured reactions were stopped by the addition of 0.5 M H_2_SO_4_ and OD was measured at 450 nm.

### Peptides and antibodies affinity purification of antibodies

DBL5ε of 3D7 PFL0030c *var2csa* sequence (Genbank accession number. XM_001350379) was used to design peptides. A library of 23 peptides (70% purity) each consisting of 20 amino acids and having an overlap of 6 amino acids was synthesized (Sigma Genosys). All peptides had a free amine at the N- and a free acid at the OH-terminus. ELISA was carried out on plates coated with 5 µg/ml of each peptide. VAR2CSA antibodies reactivity against those peptides was measured using Senegalese pregnant women plasma pool 1∶100 (pool was obtained with n = 30 multigravidae plasma) and Beninese pregnant women plasma pool 1∶100 (pool was obtained with n = 30 multigravidae plasma). Plasma samples from Unexposed French men (n = 8) and pregnant women (n = 16) were used as negative controls.

The two peptides (P4: _2037_RRQLCFSRIVRGPANLRNLK_2056_ and P13: _2163_SWCTIPTTETPPQFLRWIKE_2182_) which reacted with malaria exposed pregnant women plasma pool were used for affinity purification of antibodies. This was done using HiTrap NHS-activated HP columns (GE Healthcare) according to the manufacturer's instructions. In brief, 1 mg of each synthetic peptide was dissolved in coupling buffer 0.2 M NaHCO_3_, 0.5 M NaCl (pH 8.3), and applied to the 1 ml column previously equilibrated with 3×2 ml of ice-cold 1 mM HCl. After coupling, the columns were washed alternating 0.5 M ethanolamine, 0.5 M NaCl (pH 8.3) and 0.1 M acetate, 0.5 M NaCl (pH 4), followed by a final wash with PBS (pH 7.4). One ml of Senegalese pregnant women plasma pool was diluted in PBS (1∶1), filtered through a 0.45-µm filter and applied to the column at a flow rate of 1 ml / min. After washing the column in 7 ml PBS, affinity-bound antibodies were eluted in fractions with a total volume of 3 ml of 0.1 M glycine-HCl (pH 2.8) and neutralized in 1 M Tris (pH 8). The specificity of the purified antibodies was tested in ELISA against the peptide used for affinity purification.

### Antibody recognition of surface VAR2CSA

*P. falciparum*-IEs collected *ex vivo* from the placenta of Beninese women were used without additional *in vitro* culture. Flow cytometry was used to test the reactivity of the antibodies against either the P4 or P13 peptides with parasite isolates, as described elsewhere [34]. Briefly, mature parasites (four placental isolates) were enriched to contain >75% PE at late-stage trophozoite and schizont stages by exposure to a strong magnetic field. Aliquots of ∼2×10^5^ PE were labeled by ethidium bromide and sequentially exposed to 20 µl human purified IgG (∼0.2 µg IgG) and 1 µl goat anti-human IgG-FITC (Sigma). Data were acquired using FACS Calibur (BD Biosciences, Franklin 10 Lakes, NJ). All samples relating to a particular parasite isolate were processed and analyzed in a single assay.

### Interaction properties of the recombinant DBL5ε proteins

Binding to CSPG (decorin D8428, Sigma-Aldrich) was performed mainly as described elsewhere [35]. Briefly falcon plates (351172, Becton Dickinson) were coated with either 5 µg/ml of CSPG in PBS or with 1% BSA in PBS for background measurement (overnight at 4°C). Following coating, the wells were blocked with TSM binding buffer (20 mM Tris–HCl, 90 mM NaCl, 2 mM CaCl_2_, 2 mM MgCl_2_, 0.05% Tween-20 and 1% BSA, pH 7.4 at 25°C) at room temperature for 6h. A dilution series (0.4–40 µg/ml) of the DBL5ε recombinant domains in TSM binding buffer was added in each well and incubated overnight at 4°C with gentle shaking. After washing three times in TSM washing buffer, 100 µl of anti-His tag-HRP antibody diluted 1∶3 000 in binding buffer was added to each well and incubated for 2h at room temperature. The assay was finalised with three washes and developed using 100 µl per well of TMB substrate for 30 min. Absorbance was measured at 450 nm after quenching the reaction with 100 µl of 0.5 M H_2_SO_4_.

Inhibition of recombinant domain binding to CSPG was performed mainly as the above described ELISA analysis, but using a constant protein concentration (5 µg/ml) pre-mixed with increasing amounts of soluble CSA (0.5–400 µg/ml).

### *In silico* analyses of VAR2CSA sequences from field isolates

#### Multiple alignment

Initially a master data file was created, containing sequence ids, experimental parameters (where available) and unaligned sequences. The DBL5ε were aligned using ClustalW2 [36] with default options. The resulting alignment was inspected and manually adjusted. Aligned sequences were then inserted in the master file.

#### Evaluation of system diversity by calculation of Shannon entropy

The Shannon entropy [37] was calculated for each position in the multiple alignment as:
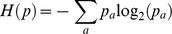
Briefly on values of *H*: *H* = 0: Complete conservation, only one residue present at the given position. 0<*H*≤1: Considered highly conserved. 1<*H*≤2: Considered conserved. 2<*H*≤4.3 considered variable. The calculated Shannon Entropy per multiple alignment position was subsequently depicted.

#### Homology modeling

DBL5ε homology models were created by submitting the multiple alignment to the HHpred server [38]. Best hit was chosen based on an evaluation of score and structure resolution (VAR2CSA DBL3x domain, PDB ID: 3bqk) [26]. One primi- and one multigravidae representative sequence were chosen and submitted individually to HHpred. The resulting models were loaded into PyMOL [39] and aligned for visual analysis of structural impact of motifs. The models were validated by submission to the ProQ server [40]. Likewise was a model of DBL5ε-3d7 created for mapping purposes.

#### Mapping of sequence variability

The sequence variability was mapped onto a homology model of DBL5ε-3d7 by submission to the H2PDB server [41]. The resulting pdb-file was loaded into PyMOL and variability was visualised by heat-map colouring (colour by b-factor).

#### SigniSite analysis

A statistical *In silico* analysis of the multiple alignment was performed using the SigniSite server [23]. Briefly: The SigniSite server performs a non-parametric statistical evaluation of the distribution of each residue at each position, aiming at identifying any significant association with a sequence associated numerical parameter, specified at submission. As a prerequisite for submission to SigniSite is the association of a numerical parameter to each sequence, sequence files were created for each numerical parameter containing the DBL5ε sequences and the associated numerical parameter (where available). Numerical isolate parameters were: Maternal age at delivery [year(s)], Concentration of parasites in peripheral blood of the mother [/µL], Concentration of parasites in the placental blood [/µL], Parity, Birth weight [g], CSA binding density [mean/mm^2^], CSPG binding density [mean/mm^2^]. Some of the women were infected with more than one parasite and thus some isolates contain more than one sequence. It should be noted that (i) numerical values associated with a particular isolate were assigned to all the sequences identified in that particular isolate and (ii) not all parameters were available for all sequences, if no parameter was available, the sequence was excluded from evaluation. As SigniSite performs multiple testing, it was imperative to reduce the number of tests performed prior to submission. This was done in two steps: (i) Exclusion of all positions in the multiple alignment with *H* = 0 (If just one residue is present at a given position, no significant distribution is possible). (ii) Evaluating only the top 15% most variable positions as estimated by the entropy calculation (It is more likely to identify a significant distribution at the most variable positions). Following this, the before mentioned sequence files were reduced to only contain the positions selected for testing. The sequence files were subsequently submitted to evaluation by SigniSite with the following settings: Significance threshold = 0.05, Correction for multiple testing using the Bonferroni single-step, Consider values given in fasta header and Choose decreasing order. The normal distributed Z-scores were converted to p-values by standard method.

### Statistical analysis

Comparison of anti-VAR2CSA antibodies levels between groups was tested by nonparametric Mann-Whitney test. Correlations were examined by use of Pearson's test. The chi^2^ test was used to examine differences between categorical variables. The Fisher's exact test was used to evaluate significance when analysing motifs and parasite expressing specific motifs identified. The significance limit was P<0.05. When evaluating DBL5ε sequences containing Q_303_ vs. E/K_303_, population means, with respect to placental parasites CSA/CSPG binding, were calculated and a two sample t-test was applied to test if differences in means were significant (p<0.01).

## Supporting Information

Figure S1 Multiple alignment of parasite isolates VAR2CSA DBL5ε sequences. cDNA from 40 placental parasites isolates (39 placental isolates from Senegal and one from Tanzania) were amplified, cloned, and sequenced. Sequence ids are given at the far left. The Tanzanian isolate was isolate 748 (sequences 748_1/2a and 748_1/2b) corresponding to the DBL5ε domain amplified in this isolate. The remaining sequences correspond to those obtained in isolates from Senegal. The remaining CYK are Senegalese isolates. The CYK suffix corresponds to the placenta id from which the isolate was extracted. The DBL5ε and ID5 highly conserved (blue, Shannon entropy 0≤H≤1), conserved (green, 1<H<1.5), and relatively variable (red, 1.5≤H≤2) blocks, are indicated. The 15% most variable positions were selected and marked with “x”.
(0.11 MB PDF)
