# Supplementary figures and images for: Insight into Antigenic Diversity of VAR2CSA-DBL5ε Domain from Multiple Plasmodium falciparum Placental Isolates
Source: PLoS One. 2010 Oct 1;5(10):e13105. doi: 10.1371/journal.pone.0013105 (PMC2948511; doi:10.1371/journal.pone.0013105)

[illegible]

DBL58

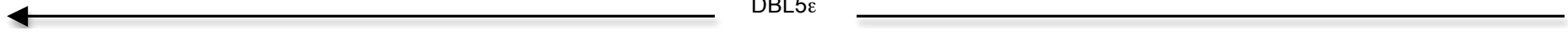

Supplement: Figure S1 — Multiple alignment of parasite isolates VAR2CSA DBL5ε sequences. cDNA from 40 placental parasites isolates (39 placental isolates from Senegal and one from Tanzania) were amplified, cloned, and sequenced. Sequence ids are given at the far left. The Tanzanian isolate was isolate 748 (sequences 748_1/2a and 748_1/2b) corresponding to the DBL5ε domain amplified in this isolate. The remaining sequences correspond to those obtained in isolates from Senegal. The remaining CYK are Senegalese isolates. The CYK suffix corresponds to the placenta id from which the isolate was extracted. The DBL5ε and ID5 highly conserved (blue, Shannon entropy 0≤H≤1), conserved (green, 1<H<1.5), and relatively variable (red, 1.5≤H≤2) blocks, are indicated. The 15% most variable positions were selected and marked with “x”. (0.11 MB PDF) [file pone.0013105.s001.pdf]
